# Research progress of the detection and analysis methods of heavy metals in plants

**DOI:** 10.3389/fpls.2024.1310328

**Published:** 2024-01-31

**Authors:** Shuang He, Yuting Niu, Lu Xing, Zongsuo Liang, Xiaomei Song, Meihai Ding, Wenli Huang

**Affiliations:** ^1^ College of Pharmacy, Shaanxi University of Chinese Medicine, Xianyang, China; ^2^ College of Life Sciences and Medicine, Key Laboratory of Plant Secondary Metabolism and Regulation in Zhejiang Province, Zhejiang Sci-Tech University, Hangzhou, China; ^3^ Key Laboratory of “Taibaiqiyao” Research and Applications, Shaanxi University of Chinese Medicine, Xianyang, China; ^4^ Management Department, Xi’an Ande Pharmaceutical Co; Ltd., Xi’an, China

**Keywords:** heavy metals, plants, detection methods, analytical methods, technology

## Abstract

Heavy metal (HM)-induced stress can lead to the enrichment of HMs in plants thereby threatening people’s lives and health via the food chain. For this reason, there is an urgent need for some reliable and practical techniques to detect and analyze the absorption, distribution, accumulation, chemical form, and transport of HMs in plants for reducing or regulating HM content. Not only does it help to explore the mechanism of plant HM response, but it also holds significant importance for cultivating plants with low levels of HMs. Even though this field has garnered significant attention recently, only minority researchers have systematically summarized the different methods of analysis. This paper outlines the detection and analysis techniques applied in recent years for determining HM concentration in plants, such as inductively coupled plasma mass spectrometry (ICP-MS), atomic absorption spectrometry (AAS), atomic fluorescence spectrometry (AFS), X-ray absorption spectroscopy (XAS), X-ray fluorescence spectrometry (XRF), laser ablation-inductively coupled plasma-mass spectrometry (LA-ICP-MS), non-invasive micro-test technology (NMT) and omics and molecular biology approaches. They can detect the chemical forms, spatial distribution, uptake and transport of HMs in plants. For this paper, the principles behind these techniques are clarified, their advantages and disadvantages are highlighted, their applications are explored, and guidance for selecting the appropriate methods to study HMs in plants is provided for later research. It is also expected to promote the innovation and development of HM-detection technologies and offer ideas for future research concerning HM accumulation in plants.

## 1 Introduction

In recent years, the HM contents of soil, water, and air have enhanced manifold due to rapid development and industrialization (Vareda et al., 2019). They are classified as essential nutrients like Fe, Zn, Cu, Ni, Mn, etc.; and non-essential nutrients like As, Hg, Cr, Pb, Cd, and Ag, based on the requirements of these elements by plants to fulfill its life cycle. HMs are mainly uptaken via the roots in the soil, while HMs in the atmosphere through the leaf surface which then accumulate in plants*. Cedrus deodara* and *Cupressus arizonica* absorb HMs through their leaves (Liang et al., 2017; Cesur et al., 2021) and those of *Nerium indicum*, *Pittosporum tobira*, and *Platanus acerifolia* through trichomes, stomata, or the mucous layer (Liang et al., 2017). The excessive accumulation of essential elements can negatively impact plants; that of Fe can cause the buildup of reactive oxygen species (ROS), damage to lipids and proteins, impairment of cell structure, leaf chlorosis, and other adverse effects (Kobayashi et al., 2019; Ahammed et al., 2020), while that of Zn can lead to nutritional imbalance, production of ethylene, stunted growth and chlorosis (Islam et al., 2014). Non-essential elements like As, Cd, Hg, and Pb, are called “toxic HMs” as they adversely affected plants when their concentrations exceeded certain levels (Wang et al., 2020; Rahman et al., 2022). These elements affected photosynthesis and respiration, damaged the structure and function of stomata, increased oxidative stress, modulated the uptake of mineral nutrients, caused DNA breaks and enzyme inactivation, and other serious adverse effects (Jaiswal and Verma A, 2018; Genchi et al., 2020; Guo et al., 2023). The toxicological impacts of common HMs on plants are summarized in **Table 1**. HMs are absorbed by the body via the food chain and are accumulated in vital organs like the liver, heart, brain, and kidneys, which disrupted their normal functions and interfered with various metabolic processes (Fu and Xi, 2020). HMs are absorbed by the body via the food chain and are accumulated in vital organs like the liver, heart, brain, and kidneys, which disrupted their normal functions and interfered with various metabolic processes (Kiran et al., 2022). Excessive accumulation of As can lead to skin diseases, diabetes, neurological complications, and impaired liver and kidney function (Kiran et al., 2022). The harmful repercussions of Hg are predominantly embodied as inattention, blurred vision, unstable walking, pneumonia, pulmonary edema, lung injury, and other symptoms. It can also be toxic to fetuses, resulting in fetal blindness, intellectual disability, speech impairment, and brain damage (Kiran et al., 2022).

**Table 1:** the toxicological impacts of common HMs on plants.

Heavy metals	Adverse impacts	References
Essential HMs		
Fe	Accumulation of reactive oxygen species, damage to lipids and proteins, damage to cell structure, leaf chlorosis	(Kobayashi et al., 2019; Ahammed et al., 2020)
Zn	Leaf fading, slow growth, nutrient imbalance, reduced photosynthesis, accumulation of reactive oxygen species, causing oxidative stress	(Islam et al., 2014; Rukhsar Ul et al., 2023)
Cu	Inhibition of plant growth, oxidative damage, nutrient imbalance, reactive oxygen species accumulation, reduced photosynthesis, leaf edge curling or greening	(Kohatsu et al., 2021; Yuan et al., 2023)
Ni	Disrupt cell metabolism, damage cells, break down chloroplast structure, decompose chlorophyll and cystoid membrane, disrupt water balance and nutrient relationship, accumulate reactive oxygen species	(Ahmed et al., 2023)
Mn	Inhibits plant growth, leaves crumpled and shriveled	(El-Jaoual and Cox, 1998)
Non-essential HMs		
Cd	Slow growth, oxidative damage, chlorophyll degradation, reduced photosynthesis, reactive oxygen species accumulation, stomatal opening and closing, nutrient distribution imbalance, altered water balance, senescence or death	(Tunçtürk et al., 2023; Yan et al., 2023)
Pd	Inhibits seed germination, slows plant growth, disturbs water balance and nutrient relationships, interferes with enzyme activity and stomatal closure, and delays carbon metabolism	(Zulfiqar et al., 2019)
Hg	Inhibits antioxidant enzyme activity and photosynthesis, inhibits plant growth, disrupts chlorophyll synthesis, and destroys chloroplast ultrastructure	(Tang et al., 2023)
As	Induces oxidative stress, damages cells and inhibits plant growth, decreases germination, reduces crop yield, and reduces carbon metabolism	(Souri et al., 2020; Banerjee et al., 2023)
Cr	Affects seed germination, slows root and tip growth, inhibits cell cycle and nitrogen assimilation, accumulates reactive oxygen species, inhibits photosynthesis, leaves chlorosis and necrosis	(Ali et al., 2023)
Aluminum (Al)	Nutritional imbalance, inhibition of photosynthesis, disruption of cellular organization, effects on protein metabolism, disruption of enzyme activity	(Zhang et al., 2014; Yang et al., 2015; Singh et al., 2017; Shetty et al., 2021)

In plants, the roots are the main organs that absorb various nutrients and non-essential elements. Roots act as the first barrier for the entry of HMs, and most plants retain these HMs in their roots to block their transfer into the aerial parts, thereby reducing the toxic effects. Therefore, root retention is significant for plant detoxification mechanisms (Ghuge et al., 2023). Additionally, certain plant species like *Arabis paniculata, Celosia argentea*, and *Corydalis davidii* accumulate HMs to a greater extent in their aboveground parts compared to the underground parts (Li et al., 2018). Due to the robust transport capacity of certain plants, the HM content in the aboveground parts is more than ten-fold higher than that in the underground parts, and the everyday life activities are not affected, making them hyperaccumulators (Shi et al., 2023). Hyperaccumulators can eliminate, reduce, or stabilize the contents of HM pollutants included in soils thus remediating HM-polluted soil. After entering its roots, the HMs are primarily localized in the vacuoles and cell walls (Cobbett, 2000; Yin et al., 2015). The cell wall is rich in pectin, hemicellulose, cellulose, lignin, and polysaccharides (Lao et al., 2023). These negatively charged biopolymers bind to HM ions; the enzymes associated with lignin biosynthesis promote cell wall synthesis and thickening, enriching HMs in the cell wall (Keyster et al., 2020; Chen et al., 2022; Lao et al., 2023). HMs that traverse the cell wall are internalized into the vacuole through ATP-dependent transporters, where they accumulate by chelating with phytochelatins (PCs) (Keyster et al., 2020; Khan et al., 2020). Therefore, compartmentalization in cell walls and vacuoles is crucial for plant detoxification mechanisms. Plants have not developed any carriers and channels specific for non-essential elements during the evolutionary process, so these elements mainly rely on the absorption pathway of mineral elements to enter plants (Andresen and Küpper, 2013; Shahid et al., 2017). HM ions enter the plant root with the assistance of nutrient element ion channels (such as Ca^2+^ and K^+^ channels) and transport proteins present in the plasma membrane. Many transporters related to HMs, including Zn-regulated transporter, iron-regulated transporter-like proteins (ZIPs), HM-associated domain (HMA), iron-regulated transporter (IRT), natural resistance-associated macrophage protein (NRAMP), yellow stripe 1-like family (YSL), etc. have been identified (Li et al., 2017a; Leng et al., 2020; Kaur et al., 2021).

The food chain is the primary means through which humans are exposed to HMs, making the control of HM accumulation in food crops, pulses, vegetables, fruits, and medicinal plants particularly crucial. Understanding and investigating in plants the biological and physiological processing involving the transport, absorption, accumulation, and detoxification of HMs can facilitate the cultivation of edible and medicinal plants with low levels of HMs and enable the utilization of hyperaccumulators for remediation of HM-contaminated soils. To understand these mechanisms, specific instruments and technical methods are needed to obtain information regarding spatial and subcellular distribution, chemical morphology, uptake, and translocation pathways of HMs. The content of HMs can be analyzed using techniques for instance inductively coupled plasma mass spectrometry (ICP-MS) and atomic absorption spectrometry (AAS); chemical form by atomic fluorescence spectrometry (AFS) and X-ray absorption spectroscopy (XAS), respectively; spatial distribution through X-ray fluorescence spectrometry (XRF) and laser ablation-inductively coupled plasma-mass spectrometry (LA-ICP-MS); and the dynamics of uptake and transport by non-invasive micro-test technology (NMT). Moreover, “omics” and molecular biology approaches contribute to understanding the molecular mechanisms underlying plant-HM interactions, facilitating further exploration of HM-induced stress.

In recent years, the accumulation, chemical form, spatial distribution, and uptake and translocation of HMs by plants have received extensive attention. However, a complete overview of the research techniques and methods employed for gaining the information mentioned above is lacking. Therefore, this paper reviews recent advances in detecting and analyzing HMs in plants in three sections. The first section deals with the background of stress caused by HM pollution and introduces the distribution characteristics, forms of existence, and toxic effects of HMs. The second systematically discusses the principles, advantages, and applications of the frequently used detection and analytical techniques for different HMs. Finally, the current state and challenges of the various technologies are summarized, and prospects for development are projected. This review is expected to facilitate the selection of optimal research methods related to HMs in plants and provide ideas for developing and improving these technologies.

## 2 HM contamination in plants

### 2.1 Sources of HMs in plants

The three main origins of HMs in plants are soil, water, and air pollution (Nagajyoti et al., 2010; Rai et al., 2019). Inappropriate disposal of industrial waste, automobile exhaust emissions, mining, extraction and smelting, energy and fuel manufacturing, and other human activities can result in HM pollution (Wu et al., 2022). HM content is highest in water ecosystems and soils, with only a minuscule proportion in the atmosphere as vapor or particles. HMs are uptake by the roots via surface water and soil and then stored in the cell walls and vacuoles; additionally, they are absorbed and enriched in leaves from the atmosphere, which affects growth and development (Nadeem et al., 2010; Rai et al., 2019; Qin et al., 2021). Carrots, sweet potatoes, and other root vegetables are predominantly contaminated by HMs in the soil (Cobbett, 2000; Zhou et al., 2016; Cesur et al., 2021), while *C. arizonica, Pinus sylvestris L.*, and *Ficus elastica* primarily through atmospheric pollution (Zwolak et al., 2019; Alaqouri et al., 2020; Ghoma et al., 2022).

### 2.2 HMs in plants

**Figure 1:**
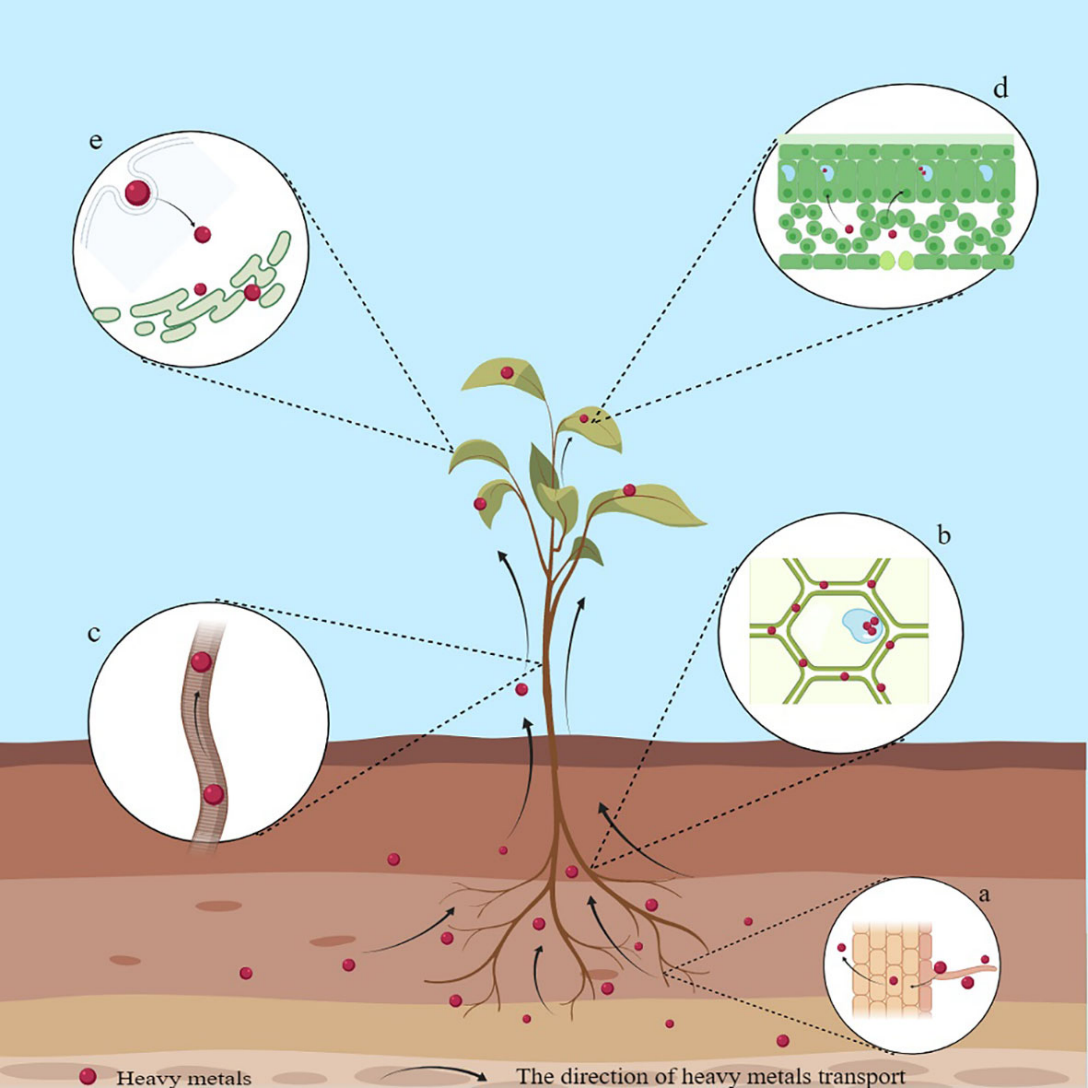
Schematic diagram of the mechanism of HM accumulation in plants. **(A)** Accumulation of HMs on the surface of the root system; **(B)** Accumulation of HMs in cell walls; **(C)** Root-to-shoot translocation; **(D)** Accumulation of HMs in leaves; **(E)** Endocytosis.

HMs are mainly selectively absorbed or diffused from the soil into plants through roots, and H^+^ coupled carrier proteins or transporters are critical for the entry of HMs into the roots (DalCorso et al., 2019). For example, plants can absorb Cd through Ca^2+^ and K^+^ channels, while transporters, including ZIP, HMA, YSL, NRAMP, and IRT, are involved in the various processes of Cd absorption and transport (Andresen and Küpper, 2013; Yin et al., 2015; Shahid et al., 2017). After entry through the roots, HMs travel through the cell membrane into the cytoplasm or combine with the cell wall. Most of the absorbed HMs accumulate directly in the roots or are loaded into the xylem by transporter proteins and then, through the xylem, are translocated to the aerial part and accrue in foliage (Yang et al., 2022) (**Figure 1**). The accumulation levels of specific HMs are generally > 100 mg kg^-1^ for Cd; 1000 mg kg^-1^ for Pb, Co, Ni, etc.; and 10,000 mg kg^-1^ for Zn and Mn (Shi et al., 2023). For example, the hyperaccumulators of Pb include *A. paniculata*, *Isachne globosa*, and *Pogonatherum crinitum* (Kaur et al., 2021; Shi et al., 2023); of Mn include *C. argentea*, *Polygonum lapathifolium*, and *Schima superba* (Kaur et al., 2021; Shi et al., 2023); of Cd include *Sedum alfredii* Hance and *Solanum nigrum* L (Al-Huqail, 2023; Hu et al., 2023).; of As include *Pteris vittata* L (Zhang et al., 2022).; and of Zn include *C. davidii*, *Picris divaricata*, and *Viola baoshanensis* (in their aerial parts) (Kaur et al., 2021; Shi et al., 2023). At the subcellular level, HMs are primarily distributed in the cell walls and vacuoles, thereby compartmentalizing them, reducing their transfer to other parts, and minimizing toxicity (Corso, 2020; Yang et al., 2022). Vacuoles being non-physiologically active organelles, HMs, upon entry into the vacuoles, are isolated, passivated, and precipitated, thereby reducing their toxicity (Corso, 2020; Yang et al., 2022). Binding sites such as -COOH, -SH, and -OH provided by the cell wall can adsorb HM ions, better defining the role of the cell wall in accumulating metal to prevent their entry into plants (Ghori et al., 2019; Corso, 2020). The cell walls of *Boehmeria nivea* L. contain high levels of Cd (Chen et al., 2022). The cell walls and vesicles of the leaves in *Leersia hexandra* Swartz are the main sites of Cr(III) accumulation (Ma et al., 2022).

In plants, the chemical forms of HMs are strongly correlated with their mobility and biotoxicity (Duffus, 2002; Yang et al., 2022). As is found in distinct valence states, primarily in arsenite (AsIII) and arsenate (AsV) forms, of which AsIII is somewhat more toxic than AsV (Souri et al., 2017). Plant species, absorption mechanisms, As speciation, and soil type are critical factors that influence the speed of absorption and buildup of As through plants (Zhao et al., 2009). Moreover, the roots discriminatingly absorb particular As forms though different transporters and pathways (Gupta et al., 2011; Farooq et al., 2016). In root cells, in order to prevent As translocation to shoots, As is translocated into the vacuole in the form of AsIII or AsIII-glutathione/phytochelatin complexes or is turned into less toxic organic forms (Smita et al., 2015). In hyperaccumulator plants, AsV could be efficiently reduced to AsIII, and then mobilized from roots to aerial part (as the dominant form of As in xylem sap) (Raab et al., 2005; Su et al., 2008). Most angiosperms use a mechanism of Fe uptake based on reduction (Grillet and Schmidt, 2017). Roots can enzymatically reduce Fe by plasma membrane-bound ferric reductase/oxidase (FRO) or chemically reduce Fe by root secretion of organic compounds (Grillet and Schmidt, 2017). Using X-ray absorption near-edge spectroscopy (XANES), Cr was primarily detected as a trivalent form (less toxic than hexavalent Cr) in *L. hexandra* Swartz roots and leaves (Liu et al., 2014). High-performance liquid chromatography/inductively coupled plasma mass spectrometry (HPLC/ICP-MS) identified the various chemical forms of As, Hg, and Pb in lotus seeds including As(III), dimethyl arsenic acid (DMA), As(V), methyl arsenic acid (MMA), phosphorus mercury (PhHg), ethyl mercury (EtHg), methyl mercury (MeHg), Hg(II), TML, TEL, and Pb(II) (Zhang et al., 2020).

Under HM-induced stress, most metals will enter the root through general or specific pumps or ion channels, while the root also releases large amounts of secretions (e.g., malate, oxalate, citrate, etc.) that bind to the metal ions, concentrate them into the apoplast, or adsorb them to cell walls, stopping them reaching the cells (Ghori et al., 2019). When exposed to Al-induced stress, *Vigna umbellata* seedlings, in their late stages of growth, acclimatized to Al-related toxicity by releasing enormous amounts of citrate through their root systems (Liu et al., 2018). Using XANES, scanning electron microscopy (SEM), and synchrotron radiation (SR)-based technology, Zn in the root epidermis of *Helichrysum microphyllum* subsp. was identified to bind to organic molecules, like malate, histidine (His), and cysteine (Cys), thereby alleviating the toxicity related to excessive Zn, suggesting that *H. microphyllum* may be a potential candidate Zn-stress-tolerant plant (Boi et al., 2020). Pectin is an important constituent in cell walls and is also requisite to chelate metal ions (Ghori et al., 2019). Cd mainly existed in the pectin- and protein-bound forms in *S. nigrum* and *Koelreuteria paniculata* (Yang et al., 2018; Wang et al., 2021).

In addition, crucial ligands like PCs, glutathione (GSH), as well as metallothioneins (MTs) can chelate with HMs, activating the HM detoxification mechanisms in plants (Thakur et al., 2022). PCs are a family of proteins induced to synthesize Cys-polypeptides and are rich in them due to the presence of various metals (Ghori et al., 2019). They are common in plants, fungi, algae, and cyanobacteria (Gupta et al., 2013). PCs can utilize the thiol groups in the Cys residues to bind with Cd and sequester these complexes in vesicles through ABC transporter proteins (Ghori et al., 2019). Next, Cd can also form complexes with other proteins or compounds and be transported elsewhere through the xylem and phloem to mitigate Cd-related toxicity effectively (Ghori et al., 2019). *Arabidopsis* plants exhibited extreme sensitivity to Cd-induced toxicity when GSH and PC were deficient (Howden et al., 1995b; Howden et al., 1995a). The AtABCC1 and 2 transporter proteins from *Arabidopsis* are crucial for isolating the PC-Cd complexes within vacuoles (Park et al., 2012). Since the *OsPCS15* and *OsPCS5* genes may be associated with Cd reduction, in Park et al.’s study of rice subjected to both Cd and As stress, it was found that *OsPCS5* and *OsPCS15* were specific to Cd and As (Park et al., 2019). GSH exists in both oxidized and reduced forms, mediates the biosynthesis of PCs, its thiol groups form S-based bonds with HMs thereby sequestering them, and the complexes are transported to vacuoles for detoxification (DalCorso et al., 2008; Ghori et al., 2019). The OsCLT1 transporter protein in rice facilitated the efflux of GSH and γ-glutamyl-cysteine (γ-Glu-Cys) from plastids to the cytoplasm, mediating the biosynthesis of PCs; the mutants lacking *OsCLT1* exhibited hypersensitivity to Cd and other HMs (Yang et al., 2016). MTs are Cys-rich proteins that can bind to various HM ions such as Cd^2+^, Cu^2+^, and Zn^2+^, maintaining metal homeostasis in plants and alleviating the undesirable toxic impacts of excessive metal ions (Saeed Ur et al., 2020; Ettiyagounder et al., 2021). *PaMT3-1* from *Phytolacca americana*, overexpressed in tobacco, encoded a protein that bound with Cd^2+^ and enhanced tolerance to Cd (Zhi et al., 2020). Results from studies on tobacco indicate that overexpression of *EhMT1* can enhance Cu buildup and tolerance in root cytoplasm as well as reduce hydrogen peroxide (H_2_O_2_) production (Xia et al., 2012).

### 2.3 Biomarkers of HMs in plants

Biomonitoring refers to using biomaterials or organisms to analyze specific characteristics of the biosphere (Decou et al., 2019). The application of biomarkers for environmental biomonitoring can detect the harmful effects of pollutants at an early stage and play the role of an early warning system. Morphology, physiology, and biochemistry alter when plants are subjected to HM-induced stress as well as can be used as biomarkers (Decou et al., 2019; Krayem et al., 2021). These alterations include suppression of root development, leaf discoloration and necrosis, suppression of photosynthesis and respiration, and induction of oxidative stress (Costa et al., 2018; Decou et al., 2019; Krayem et al., 2021). Substantial research has explored the biomarkers or bioindicators of HMs in plants. An analysis of biomarkers in *Myriophyllum alterniflorum* indicated a direct correlation between malondialdehyde (MDA), α-tocopherol, and glucose-6-phosphate dehydrogenase (G6PDH) with Cu and Cd, which suggested that these three biomarkers were effective for metal exposure analyses (Decou et al., 2019). A comparison of the selected biomarkers, MT and PC in *Cystoseria indica*, revealed that PC reacted to HMs to a lesser extent than MT, limiting its use in HM biomonitoring (Sinaei et al., 2018). Three types of markers (molecular markers, resistance markers, and damage markers) appeared at different frequencies when the HM accumulating plant *Tillandsia ionantha* Planch. was exposed to Cd (Zhang et al., 2023). Most of the markers mentioned above are really consequences of HM toxicity and are usually visible after severe toxicity, Only PCs and MTs are related to metals and the others are general stress markers. In conclusion, elucidating the mode of action and potential molecular mechanisms of biomarkers for detecting HMs in plants could improve their applicability in environmental quality assessment and facilitate the development of effective biomonitoring and phytoremediation technologies (Krayem et al., 2021; Saroop and Tamchos, 2021; Esmaeilzadeh et al., 2023).

## 3 Detection and analysis techniques of HMs in plants

Robust detection tools and analytical technologies enable a holistic understanding of the mechanisms involved in the uptake, transport, accumulation and detoxification of HMs in plants. In recent years, HM-detection technology has concentrated on precision, comprehensiveness, and automation, which are crucial for regulating and decreasing the levels of HMs in plants. ICP-MS, AAS, and AFS are used to detect the contents of HMs in plants. AFS and XAS can identify the chemical forms of HMs. XRF and LA-ICP-MS can elaborate the distribution of HMs in sub-cells and tissues. NMT enables measuring the flow rate of HM ions. Omics and molecular biology techniques can analyze the molecular mechanisms through which plants interact with HMs and discover more genes related to HM resistance. The principles, advantages, and applications of these analytical techniques for detecting different HMs in plants are systematically discussed subsequently.

### 3.1 Quantitative analysis

#### 3.1.1 Inductively coupled plasma mass spectrometry

**Figure 2:**
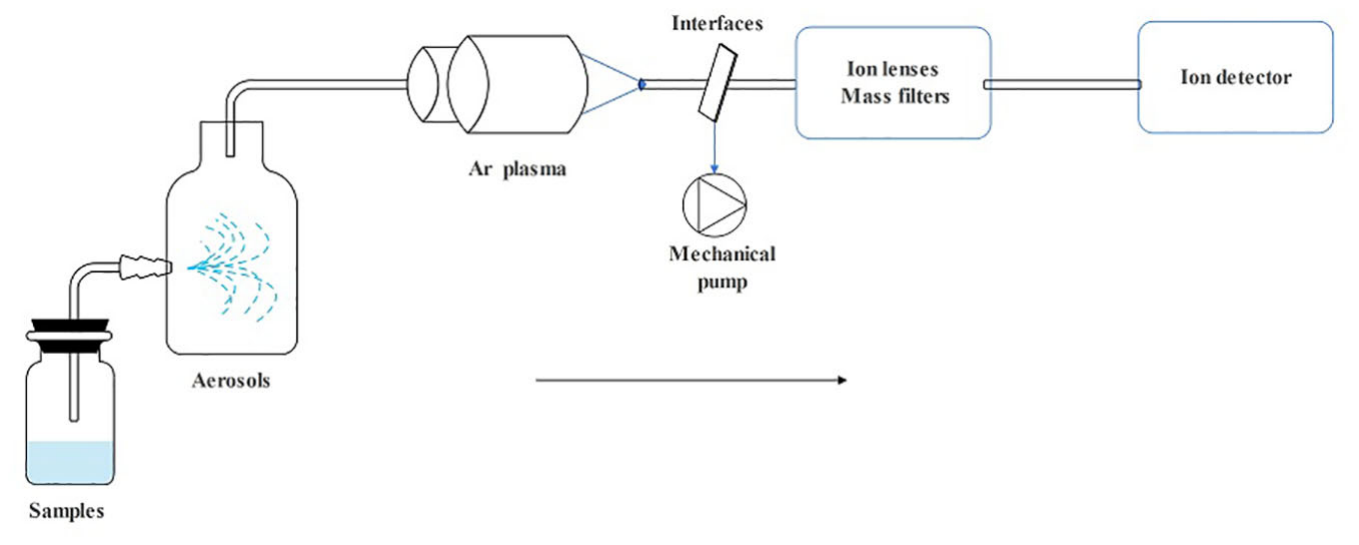
The operating principle schematic diagram of ICP-MS.

ICP spectroscopy is a method used to analyze trace metals by measuring light emission with characteristic wavelengths when the metal is exposed to an electric current. ICP-MS includes a sample introduction system, ion source, interface, ion lens, mass filter, and an ion detector (Yu et al., 2020). The details of how it works are as follows: First, the sample is drawn into the nebulizer by a peristaltic pump or self-priming to generate an aerosol of fine liquid droplets. Next, they are introduced into the central zone of the high-temperature plasma that was created by combining the energy supplied to the coil by the ICP radio frequency (RF) emitter and Ar gas. As their absorption increases, electrons are released, and cations are formed. The ion lens then focuses them through the interface, separates them based on their mass-to-nucleus ratio using a mass filter, and transports them to the detector for analysis resulting in a mass spectrum (Wilschefski and Baxter, 2019) (**Figure 2**). ICP-MS provides the advantages of being capable of identifying multiple elements, fast, sensitive, and capable of detecting minute levels. It also has a wide linear range, allows for easy control of interferences, and provides information on isotopes (Wilschefski and Baxter, 2019; Theiner et al., 2020; Penanes et al., 2022).

ICP-MS is a powerful technology with excellent capability for analyzing elements and detecting the concentration of HMs in plants at trace levels (Huang et al., 2006; Wilschefski and Baxter, 2019). Using ICP-MS, the content of Cd in the aromatic medicinal plant *Alkana trichophila* was detected to exceed the World Health Organization (WHO) standards; however, the content of other trace elements, like Al, V, and Cr, were within the acceptable range, suggesting that *A. trichophila* contains excessive amounts of Cd (Izol et al., 2023). It would depend on the concentration available in the soil (Emamverdian et al., 2018; Izol et al., 2023). The levels of four HMs: Hg, As, Cd, and Pb, in 50 medicine food homology plants were detected by inductively coupled plasma-tandem mass spectrometry (ICP-MS-MS) (Fu et al., 2018). Moreover, ICP-MS is considered the preferred way for detecting HM contents because of its advantages, like high accuracy, sensitivity, selectivity, and low detection limits. It is often used in combination with other analytical methods, for example, LA-ICP-MS, single-particle- inductively coupled plasma-mass spectrometry (SP-ICP-MS), liquid chromatography- inductively coupled plasma-mass spectrometry (LC-ICP-MS), etc., for the determination of the content and the form of multiple trace elements and isotope analysis in different types of samples and various matrix samples and simplifying the sample preparing process. Utilizing HPLC-ICP-MS, the As content in quinoa and rice was detected to exceed the food pollutant standards set by the European Commission, indicating a severe overaccumulation of As (D’Amore et al., 2023). LC-ICP-MS and ICP-MS were used to detect the As content of ten species of algae, and it was found that *Cystoseira species* and *Cystoseira fragile* had the highest total As content (Pell et al., 2013). I In addition to *Cystoseiraz*, inorganic As was dominant in the other nine algal species, indicating that ICP-MS and LC-ICP-MS provide reasonable technical support for further exploration of the As content in marine plants (Pell et al., 2013). LA-ICP-MS detected that, except *Ginkgo biloba*, Pb and Cd accumulated rapidly in four tree species including *Pinus densiflora*, *Chamaecyparis obtuse*, *Salix koreensis*, and *Platanus occidentalis*, while the distribution of Fe, Cr, Mn, and other elements exhibited changes in tree-ring patterns (Kim et al., 2020). This indicates that LA-ICP-MS can be employed to determine changes in levels of certain HMs in tree rings, providing valuable information and contributing to research in environmental pollution monitoring (Kim et al., 2020). The application of SP-ICP-MS offers a distinct opportunity to detect and study the concentration of metal particles, particle-particle and particle-matrix interactions, solubility, etc. Additionally, it allows for elemental analysis of single-celled organisms and provides isotope-related information over an extensive dynamic range of individual cells. SP-ICP-MS was utilized to detect the rapid absorption of Zn by *Lacuca sativa* L. plants grown in a medium containing ZnO nanoparticles (Wojcieszek et al., 2019). Nicotinamide formed a complex with 70% of the Zn in leaves as detected by ICP-MS and electrospray ionization-tandem mass spectrometry (ESI-MS-MS) (Wojcieszek et al., 2019). In summary, with the update and progress of technology and methods, ICP-MS has become a multifunctional and powerful platform that can determine the total elemental concentration and expand the scope of application with other technologies, providing a multidimensional perspective for solving problems related to HMs.

#### 3.1.2 Atomic absorption spectrometry

**Figure 3:**
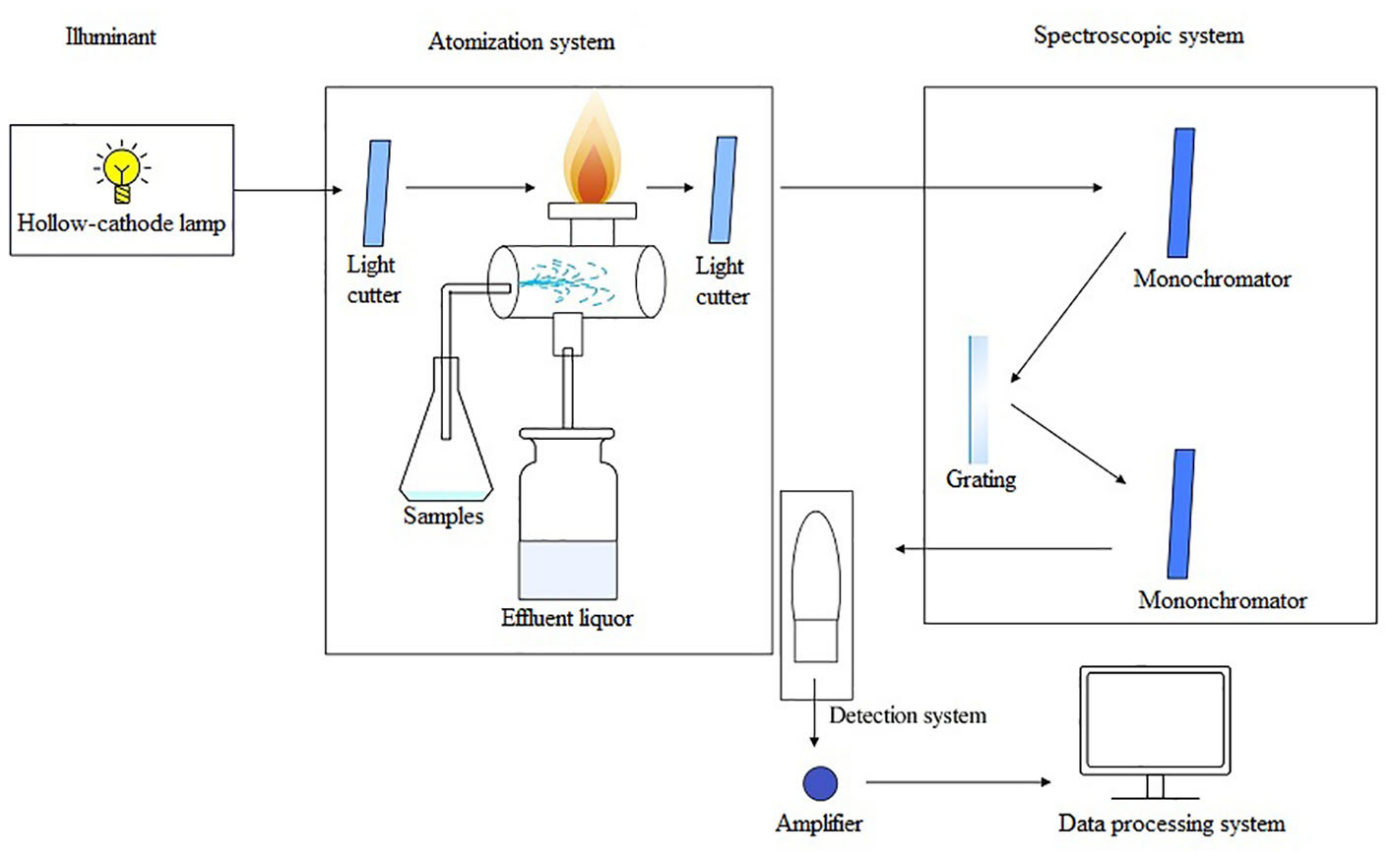
The operating principle schematic diagram of AAS.

AAS is one of the earliest commercially developed methods for the elemental analysis of HMs (Jin et al., 2020). AAS is an approach for quantitative analysis based on the absorption of characteristic spectral lines by atomic vapor produced from a substance. It comprises five main components: a light source, an atomization system, a spectroscopy system, a detection system, and a display unit. The operating principle is as follows: First, an atomizer turns the sample to be measured into an atomic vapor under high temperature. Next, when the atomic vapor is irradiated with a light source, it can absorb light radiation of a certain wavelength. After this, the spectroscopic system distinguishes between different spectral lines. Finally, the content of the element to be measured in the sample is determined according to the degree of attenuation of the light when it is absorbed (**Figure 3**). AAS possesses the advantages of high selectivity, accuracy, sensitivity, and low interferences ability. The equipment is easy to operate, enables fast analysis, and provides an extensive range of analyses (Eskina et al., 2020). Depending on the atomization device used, AAS can be categorized into cold vapor generation atomic absorption spectrometry (CVAAS), hydride generation atomic absorption spectrometry (HGAAS), graphite furnace atomic absorption spectrometry (GFAAS), and flame atomic absorption spectrometry (FAAS) (Butler et al., 2006; Yuan et al., 2011; Guo et al., 2023).

AAS is one of the very popular methods for the qualitative and quantitative analysis of HMs in plants and can be employed to measure directly the content of metal elements in the sample (Adolfo et al., 2019). For instance, 12 elements, including Ag, Cr, Cu, Ca, and Fe, were detected in the leaves of *Andrographis paniculata* using AAS (Kartha. and PP, 2021). Similar results were observed with eight HMs (e.g., Cd, Cr, and Hg) in cereals, tubers, and vegetables, as detected by AAS (Omeje et al., 2021). Although AAS effectively determined the composition and quantification of elements, its efficacy can be influenced by the extraction technique, the elements to be determined, and the plant tissue (Elik et al., 2020). For example, AAS detected that the Mn content of five plant samples was highest when extracted using a nitric acid-perchloric acid solution, indicating that different digestion methods used for preparing plant samples impacted the detection of HM contents (Bankaji et al., 2023). Therefore, proper digestion methods are crucial for the maximal extraction of specific metals from various samples (Bankaji et al., 2023). Although new HM detection techniques have been developed, AAS remains one of the most potent tools in plant elemental metal analysis and trace analysis due to its cost-effective advantages, low price, low detection limit, and good repeatability (Vinogradova et al., 2023).

### 3.2 Chemical forms analysis

#### 3.2.1 Atomic fluorescence spectrometry

**Figure 4:**
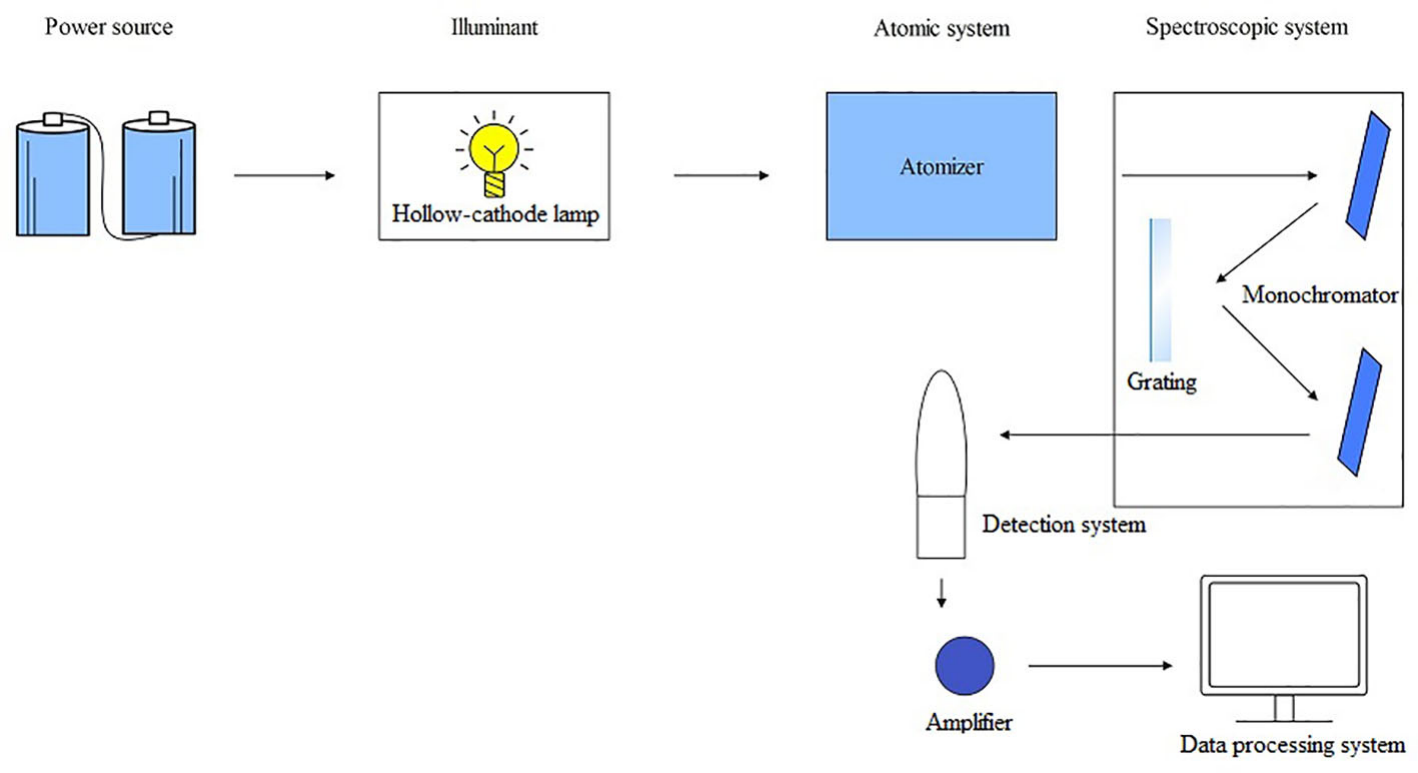
The operating principle schematic diagram of AFS.

AFS is a fast-growing technique for trace analysis and belongs to emission spectroscopy, an important branch of atomic spectroscopy. AFS consists of five parts: a light source, an atomization system, a spectroscopic system, a detection system, and a display device. The working principle of AFS is that after the sample to be analyzed is converted into atomic vapor by the atomizer, the atomic vapor absorbs light radiation at its characteristic wavelength, causing the atoms to transition from the ground state to the excited state for a duration of 8 – 10 seconds. During this process, fluorescence with either the same or different wavelength as absorption is emitted. This fluorescence is then converted into an electrical signal by a photoelectricity detector and processed into readable data by a data processing system (Costa Ferreira et al., 2019) (**Figure 4**). Atomic fluorescence is divided into three types: resonant fluorescence, non-resonant fluorescence, and sensitive light fluorescence. Among them, resonant fluorescence has the highest intensity and is the most commonly used. AFS by atomization can be further divided into cold vapor atomic fluorescence spectrometry (CVAFS) and hydride generation atomic fluorescence spectrometry (HGAFS). AFS offers advantages like high accuracy (similar to AAS), high sensitivity, simultaneous determination of multiple elements, low detection limits (including very low detection limits for Zn and Cd), simple spectral lines, minimal spectral interference, wide linear range, etc (Yogarajah and Tsai, 2015; Xia et al., 2019; Peng et al., 2022). It is a simple instrument with lower operating costs compared to AAS.

Studies have shown that AFS is often combined with chromatographic techniques to define trace and ultra-trace element levels in plants, as well as analyze their Chemical forms (Dai et al., 2019; Li et al., 2021; Guo et al., 2023). Cheng et al. used dual-frequency ultrasonic-assisted enzyme digestion (UDUE) combined with liquid chromatograph-hydride generation atomic fluorescence spectrometry (LC-HGAFS) technology to detect the content and form of As elements in *Paeoniae Raxix rubra, Angelica Sinensis, Codonopsis pilosula*, and *Salvia* (Cheng et al., 2020). The results indicate that inorganic As is present in both trivalent and pentavalent forms in four medicinal plants (Cheng et al., 2020). Deng et al. utilized the method of high-performance liquid chromatography and hydride generation atomic fluorescence spectrometry (HPLC-HG-AFS) to detect various forms of As in rice that include As(V), DMA, As(III), and MMA (Deng et al., 2021). Similarly, the As forms of the three aquatic plants were mainly As(III) and As(V), which were detected using liquid chromatograph-atomic fluorescence spectrophotometry (LC-AFS) by Wang et al (Wang et al., 2021). Besides, plenty of studies have shown demonstrated that As typically exists in the form of As(V) and As(III) within plants (Jedynak et al., 2010; Budzyńska et al., 2017; Budzyńska et al., 2021). Hu et al. determined the levels of inorganic Hg in various vegetable and fruit samples using hydride generation novel ultraviolet atomization atomic fluorescence spectrometry (HG-UV-AFS). They discovered that volatile methyl Hg^+^ hydride (MeHgH) could be converted into elemental Hg vapor through UV-based atomization and KBH_4_ treatment, and they also detected total Hg (Methyl Hg^+^ (MeHg) and Hg^2+^) (Hu et al., 2018). These results showed that HG-UV-AFS effectively detected gaseous organic Hg hydride, providing a reference for analyzing HMs in their gaseous state (Hu et al., 2018). Se in garlic was determined using reversed-phase high-performance liquid chromatography and hydride generation atomic fluorescence spectrometry (RP-HPLC-HG-AFS) revealing the four forms of Se: SeMeSeCys, SeMet, Se(VI), and Se(IV) (Castro Grijalba et al., 2017). Furthermore, RP-HPLC-HG-AFS method provides technical support for the Chemical forms analysis of Se in samples of complex plants by effectively separating organic and inorganic Se using different ionic liquids as mobile phases (Castro Grijalba et al., 2017). In conclusion, AFS is an effective technique for identifying the chemical forms of HMs.

#### 3.2.2 X-ray absorption spectroscopy

**Figure 5:**
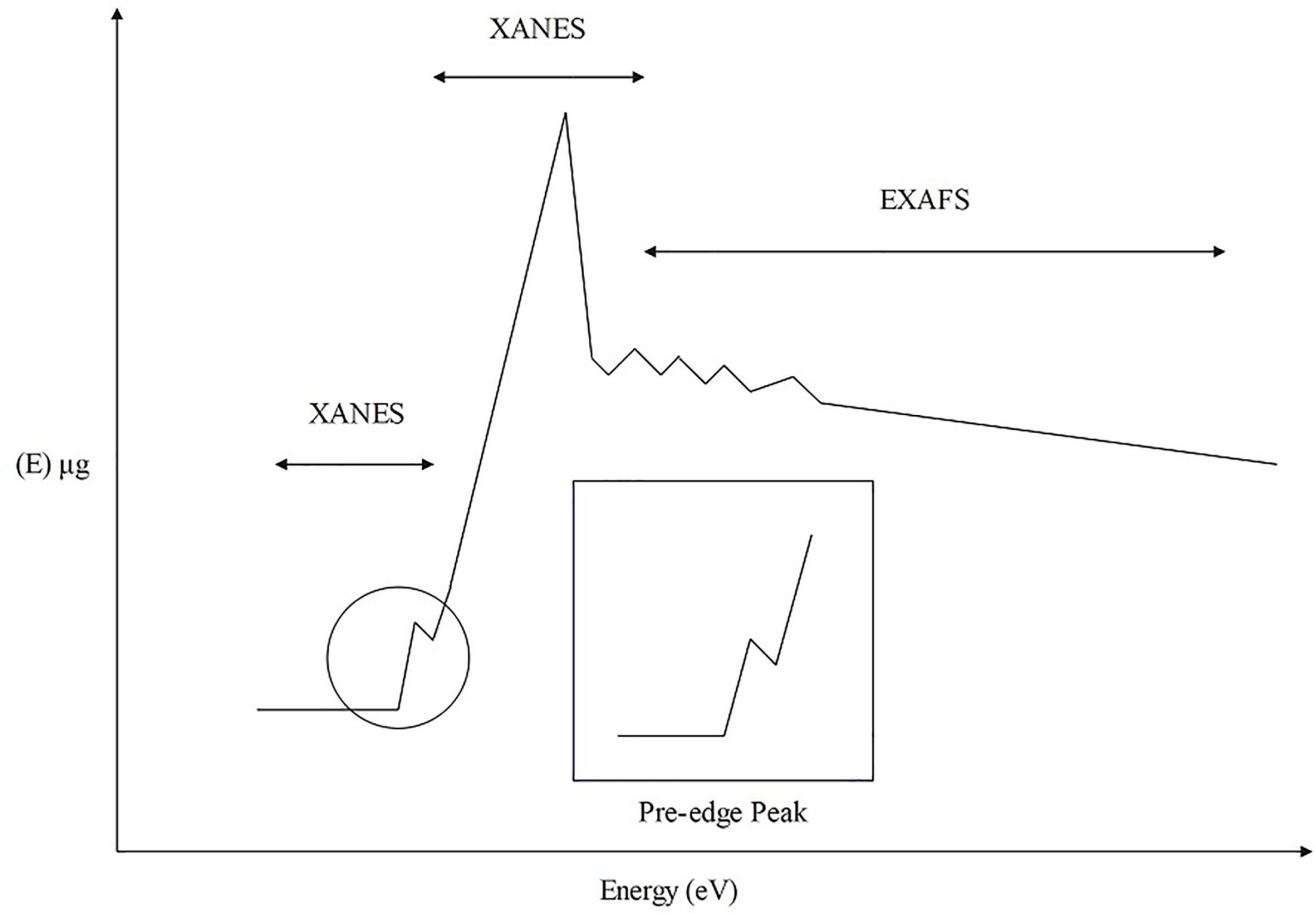
XAS structure composition diagram.

**Figure 6:**
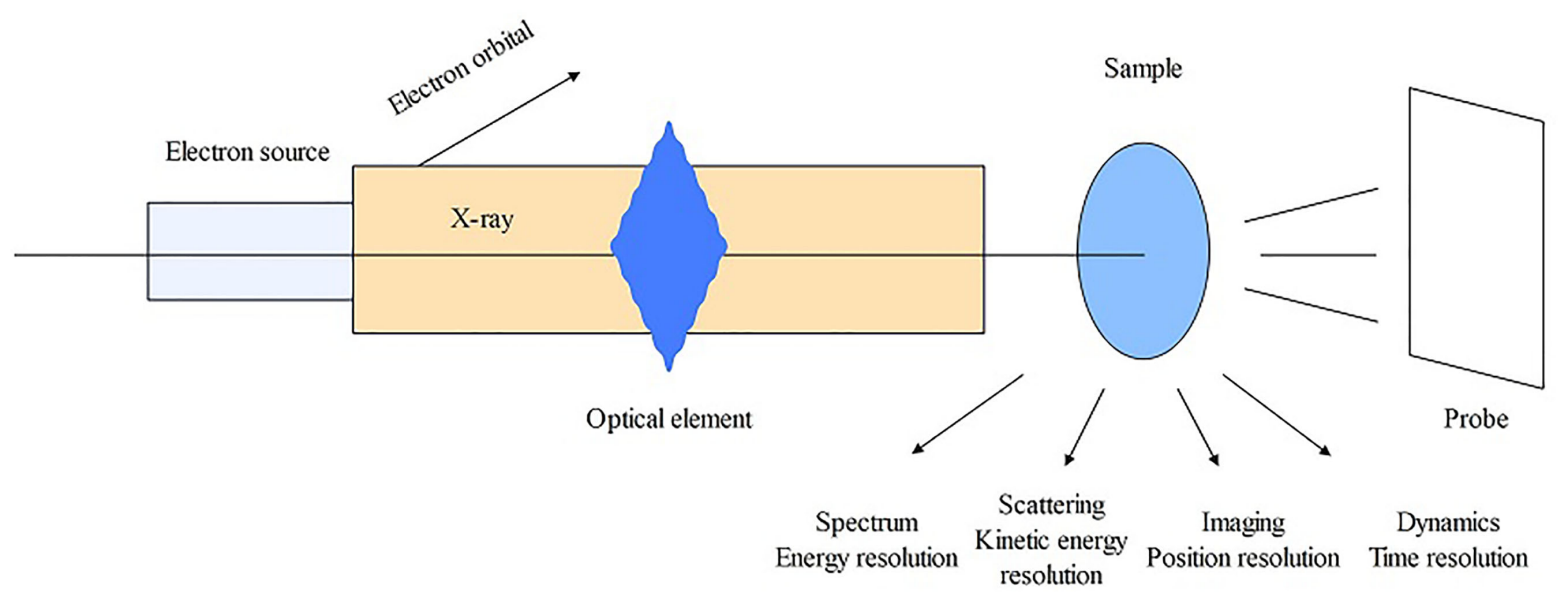
The operating principle schematic diagram of XAS.

**Figure 7:**
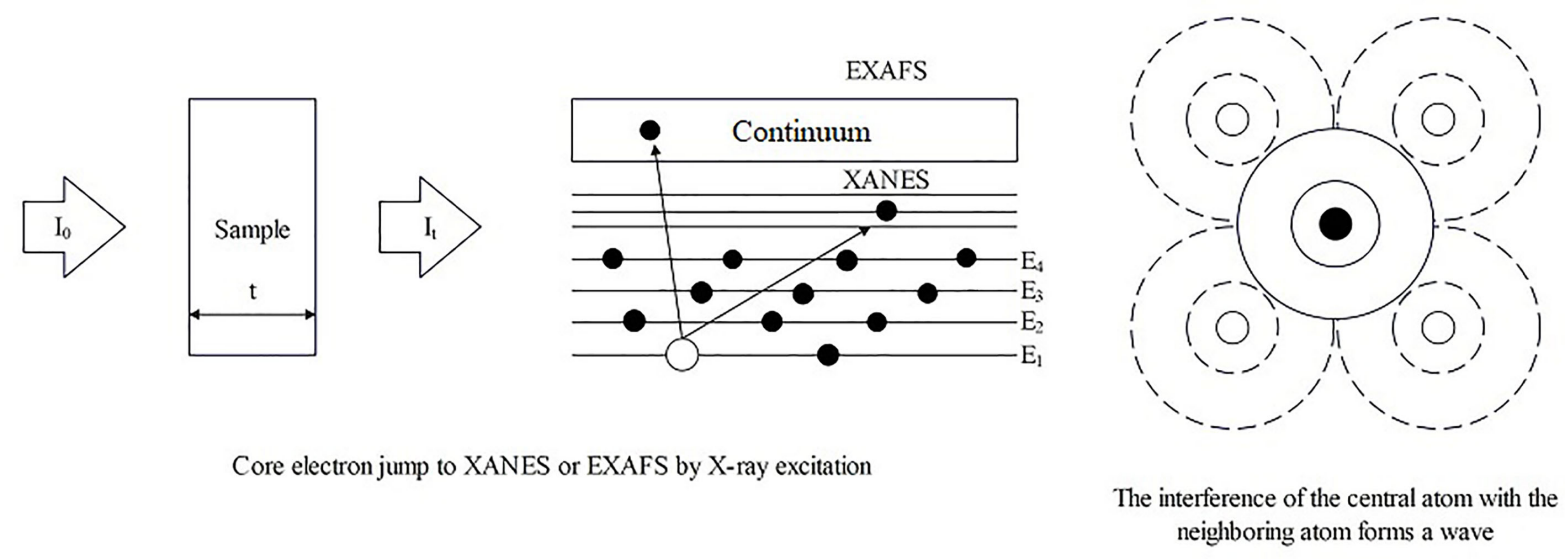
The operating principle schematic diagram of XAS.

XAS is a spectroscopic analysis approach that utilizes synchrotron X-ray sources and varies the photon energy to determine the absorption coefficient of a sample. The XAS consists of an absorption edge and a series of oscillating structures, mainly divided into two regions: the extended X-ray absorption fine structure (EXAFS) located in the 50 – 11000 eV region of the absorption edge, and the XANES located near the 50 eV region of the absorption edge (Sarret et al., 2013; Tang et al., 2023) (**Figure 5**). The XAS works by exciting the sample with X-rays, causing its core electrons to transition to either the continuum or empty orbitals (**Figures 6**, **7**). This leads to the formation of waves that scatter with the surrounding atoms (**Figure 7**). EXAFS can identify local information structures, such as bond lengths, coordination numbers, and disorder of central and coordination atoms, with a spatial resolution ranging from 0.1 – 1 pm; moreover, it is sensitive to steric structures. XANES can provide a wealth of chemical information, including near-neighbor atomic positions and chemical valence states. Meng et al. discovered that methylmercury constituted the primary form of Hg in rice (Meng et al., 2014). EXAFS can also detect the Chemical forms information of metal elements by oscillating in the EXAFS region (Hu et al., 2020). Compared to EXAFS, XANES exhibits characteristics such as subtle structural changes, a shorter acquisition time, high sensitivity to information like valence states, and faster identification of chemical types and forms of elements (Hara et al., 2023; Ren et al., 2023). XAS requires low sample amounts, does not damage the sample, and provides information on coordination atom types, coordination numbers, and atomic spacing. This facilitates the study of dynamic processes in the *in situ* electrochemical reactions and enables the accurate establishment of conformational relationships (Newville, 2014; Mastelaro and Zanotto, 2018; Tang et al., 2023).

XAS is a local structure detection technique that can be utilized to determine the distribution and morphological structural information of metal elements in plants. Sikhumbuzo et al. detected that the photon energy position of Cd overlapped with CdO in rice crops through XANES spectroscopy, and EXAFS spectroscopy revealed that CdO formed clusters in rice with a CD-O coordination number of 4.2 and a bond distance of 2.83, indicating that the main form of Cd in rice was Cd(II)-O (Kunene et al., 2020). These results indicated that XAS could function as a fundamental tool for analyzing the local atomic structure and oxidation state of Cd in rice, which helps study the mechanism of the interactions between rice plants and Cd (Kunene et al., 2020). Huang et al. detected Cr in *Coptis chinensis* Franch. using XANES and observed that Cr(VI) was reverted to Cr(III) after treatment, suggesting that toxic hexavalent Cr can be transformed into non-toxic trivalent Cr in plants (Huang et al., 2018). XANES detected the main form of Pb in *Oenanthe javanica* as Pb-ferrihydrite, which was absorbed and transported as Pb(Ac)_2_-3H_2_O (Liu and Luo, 2019). From the above studies, it can be observed that XAS enables the detection of HMs’ forms in plants, facilitating the analysis of HMs’ absorption and transport mechanisms in plants. This provides a direction for studying plant HM tolerance and offers possibilities for investigating the valency, ligand and dynamic metabolism of HMs in plants.

### 3.3 Spatial distribution

#### 3.3.1 X-ray fluorescence spectrometry

**Figure 8:**
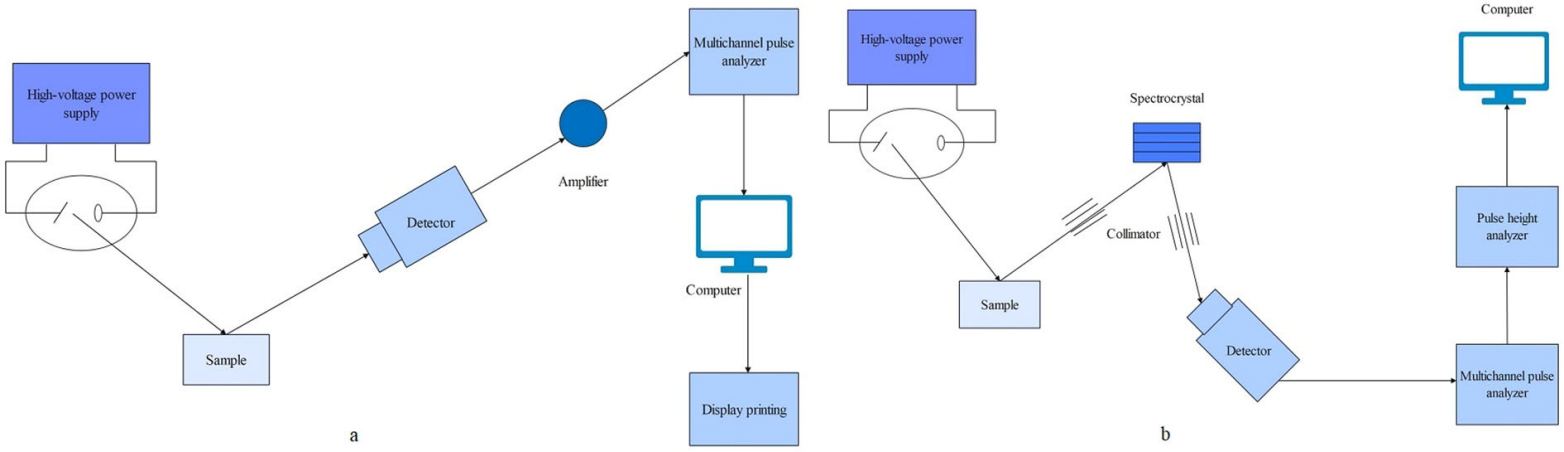
The operating principle schematic diagram of XRF. **(A)** Energy chromatograph **(B)** Wavelength chromatograph.

XRF is an analytical technique based on the characteristic X-ray emission spectrum produced by the sample (Byers et al., 2019). It is primarily composed of an X-ray generator, a spectroscopic detection system, and a counting recording system. XRF is divided into energy-dispersive X-ray fluorescence spectroscopy (EDXRF/EDX) and wavelength-dispersive X-ray fluorescence spectroscopy (WDXRF/WDX) according to the description of X-rays (**Figure 8**) (Feng et al., 2021). XRF works by using an X-ray tube to excite and irradiate the sample, causing the elements in the sample to emit characteristic X-rays. These X-rays are then collimated by a filter and detected by a probe, such as a scintillation counter, which converts them into electrical signals. After signal processing, analytical results of the elemental composition of the samples are got (**Figure 8**). XRF is a physical analysis method, primarily used for surface scanning. XRF technology can be utilized to the direct analysis of solid samples and *in situ* analysis of plant samples (Marguí et al., 2009). To ground into a fine powder plant materials for high-pressure granulation, after is the preparation of plant samples by XRF technique carries on the analysis of the most common method (Rudman, 1999). It offers advantages such as high sensitivity, fast and accurate analysis, non-destructive sampling, and simple operability (Bamrah et al., 2019). SR is an advanced light source that emits pulsed light ranging from infrared to hard X-rays, with a pulse duration ≤ one nanosecond, which is many orders of magnitude higher than traditional light sources (Hu et al., 2020). Through the interaction of synchrotron radiation with matter, technologies such as XRF and X-ray photoelectron spectroscopy (XPS) have been developed (Li et al., 2015a; Li et al., 2015b). Synchrotron radiation X-ray fluorescence spectroscopy (SRXRF) can improve the sensitivity of Zn detection to about one part per billion (ppb) (Li et al., 2015a; Li et al., 2015b).

The XRF technique can generate a distinctive set of characteristics for each element in the sample, based on the concentration and intensity of the X-ray source (McGladdery et al., 2018). Fluorescence X-ray analysis is a reliable approach for both qualitative and quantitative assessment (McGladdery et al., 2018). Real-time analysis of XRF can be utilized to chart the distribution in plant tissues of HM elements (Jones et al., 2020; Ramakrishna, 2023). Studies have shown that XRF has become the preferred approach for studying the distribution of HMs in plants (Kopittke et al., 2018; van der Ent et al., 2022) The aboveground part of *Origanum sipyleum* L were prepared into powdered samples and aqueous extracts which were detected by XRF and found to be higher in nutrients such as K, Na, and Ca (Durmuşkahya et al., 2016). Sometimes, it can also be used in combination with other technologies to enhance the predictability of XRF (Feng et al., 2021). Vigani et al. utilized XRF and ICP-MS techniques to analyze cucumber leaves, revealing that Zn was primarily distributed in chloroplasts and mitochondria, and found that the Zn content significantly increased in chloroplasts and mitochondria when Fe was deficient, indicating that iron deficiency affected the distribution of Zn in leaves (Vigani et al., 2018). SR technology offers significant advantages in terms of spatial resolution, distribution, and chemical state-related information. Microscopic X-ray fluorescence (μ-XRF) and XAS technologies, based on SR imaging technology, can be used to study the distribution of metals and metal-like elements in plants while providing valence states of elements with femtosecond sensitivity and micro/nano range spatial resolution (Vijayan et al., 2015). Zn and Cd in leaves of *Arabidopsis halleri* plants were analyzed by using μ-XRF, and the results shown that they were mainly distributed in leaf trichomes, while after treatment with 100 µM Zn and 20 µM Cd, they were primarily found in mesophyll tissues (Fukuda et al., 2020). At the cellular/tissue level, Zn is mainly distributed in mesophyll tissue, while Cd is primarily found in the vascular bundles of leaf veins (Fukuda et al., 2020). The results of XAS and synchrotron radiation microscopic X-ray fluorescence (SR-μXRF) showed that the main form of La in cucumber (*Cucumis sativus*) plants was phosphate or carboxyl complex, while the main form of Ce was CeO, and a small portion of CeO_2_ was converted into a Ce(III)-carboxyl complex (Ma et al., 2015). The accumulation of Zn in leaf tips and vacuoles, as well as the dynamic complexation of histidine with Zn in edible plants, were discovered using μXRF and XAS techniques, providing new support for understanding the dynamic characteristics of Zn Chemical forms in plants (Mishra et al., 2020). *C. chinensis* was analyzed using SR-μXRF and LA-ICP-MS techniques to identify the distribution of Cr in root and the result revealed that Cr was largely accumulated in the vascular column, and part of the cortex (Huang et al., 2018). XRF is considered a powerful technical means for qualitative and quantitative detection of element distribution in single cells of plants or algae, making it highly suitable for detecting HMs in plants. It can aid in explaining the mechanisms behind the accumulation, absorption, and detoxification of HM elements in plants.

#### 3.3.2 Laser ablation-inductively coupled plasma-mass spectrometry

**Figure 9:**
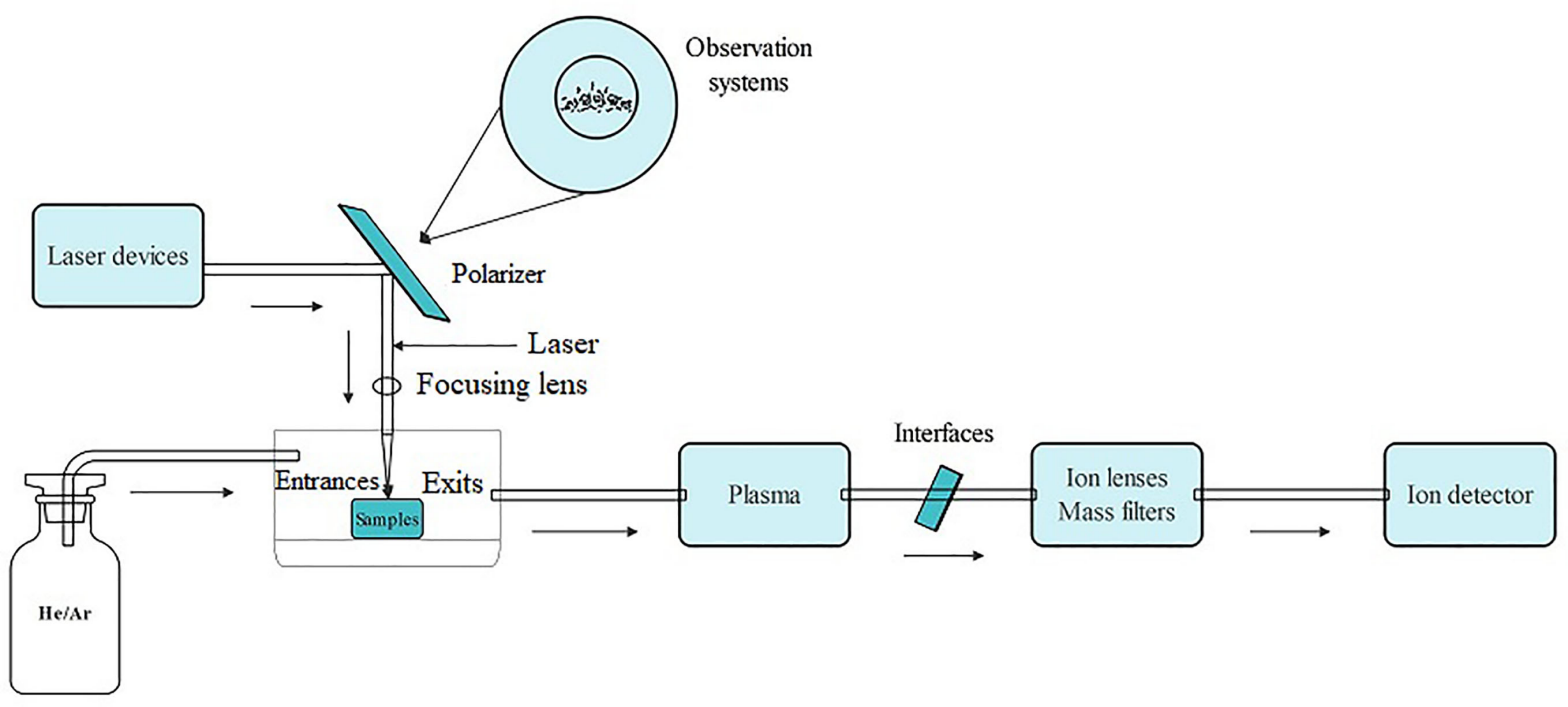
The operating principle schematic diagram of LA-ICP-MS.

LA-ICP-MS is a technique which enables rapid analysis of the distribution and content of elements in a sample at a micro-regional level, both for elemental and isotopic analysis (Pan et al., 2022). The LA-ICP-MS system is composed of three components: a laser exfoliation device (LXD), an ICP source, and a mass spectrometry detector. Among these, the LXD is composed of a laser, a beam delivery system, a sample cell, and an observation system. When a laser scans a sample, the excitation beam consisting of high-energy photons causes a photo-thermal effect that volatilizes atoms or molecules on the sample surface into particles and subsequently forms a small burrow hole, a process known as laser exfoliation (Trejos et al., 2003; Trejos and Almirall, 2005; López-Fernández et al., 2016). LA-ICP-MS works by melting and vaporizing the sample through laser ablation, and the carrier gas transports the ablated particles into the plasma (**Figure 9**). In the plasma, the particulates are ionized into positively charged ions, then analyzed by a mass spectrometry detector to identify the content and distribution of each element (**Figure 9**). During the laser scanning process, the changes in ionic strength of each element are recorded over time. This raw data is then reconstructed as an image, which is ultimately calibrated and visualized. The resulting image can be further processed using algorithms to visually represent the distribution of the elements. LA-ICP-MS can perform *in situ* analyses rapidly and in real-time on a micro-scale. It offers several advantages, including easy sample preparation, minimal contamination levels, high spatial resolution, sensitivity, low detection limits, provides relatively simple spectra while allowing for simultaneous determination of multiple elements and information about isotope ratio (Trejos and Almirall, 2005; López-Fernández et al., 2016; Nunes et al., 2016; Davison et al., 2023; Janovszky et al., 2023).

LA-ICP-MS is a robust technique that combines not only the high sensitivity and multi-element capability of ICP-MS but also good spatial resolution and laser radiation (Nunes et al., 2016). It is commonly used to directly analyze plant tissues and generate the multi-element bio-imaging of plant tissues to facilitate assess the ecological and toxicological risks of HMs to plants, and has been successfully used to analyze elemental distributions in samples such as leaves, roots, seeds, and bark (Narewski et al., 2000; Meharg et al., 2008; Shi et al., 2009; Wu et al., 2009; Yamaji and Ma, 2019). Using LA-ICP-MS imaging to detect Pb accumulation in wheat grain at maturation, it was identified the main distribution of Pb in wheat peels and seed coating, while nutrients are primarily distributed in the inner seed coating region; it was hypothesized that removing bran may be an effective way to reduce Pb levels in wheat while retaining the nutrients (Zhang et al., 2022). LA-ICP-MS detected that Fe and Zn in *Phyllostachys edulis* seeds were mainly distributed and enriched in the aleurone layer and embryo and were significantly higher than rice, asserting their suitability as a natural food enhancer (Hu et al., 2023). Thus, LA-ICP-MS provided technical support for enhancing the preservation of nutrients in *P. edulis* seeds during food processing (Hu et al., 2023). Yamaji et al. utilized LA-ICP-MS technology to establish a bioimaging scheme for various elements at the cell level of rice nodes and identified the distribution characteristics of mineral elements between the wild-type and *OsHMA2* mutant rice plants (Yamaji and Ma, 2019). K, P, Mg, and other nutrients were mainly distributed in the phloem, while Zn, Fe, and other trace elements in the vascular tissues (Yamaji and Ma, 2019). The distribution of Zn in the nodal tissues of the *OsHMA2* mutant was not markedly distinct from that of the wild type. However, the contents of Zn were lower in the nodal vascular tissue I but elevated in III compared to the wild type (Yamaji and Ma, 2019). This suggests that LA-ICP-MS is a robust and powerful method for the qualitative and quantitative mapping of mineral elements in rice node tissues, especially when combined with the functional characterization of transporter proteins (Yamaji and Ma, 2019).

### 3.4 Absorption and transport of HMs by plants

#### 3.4.1 Non-invasive micro-test technology

**Figure 10:**
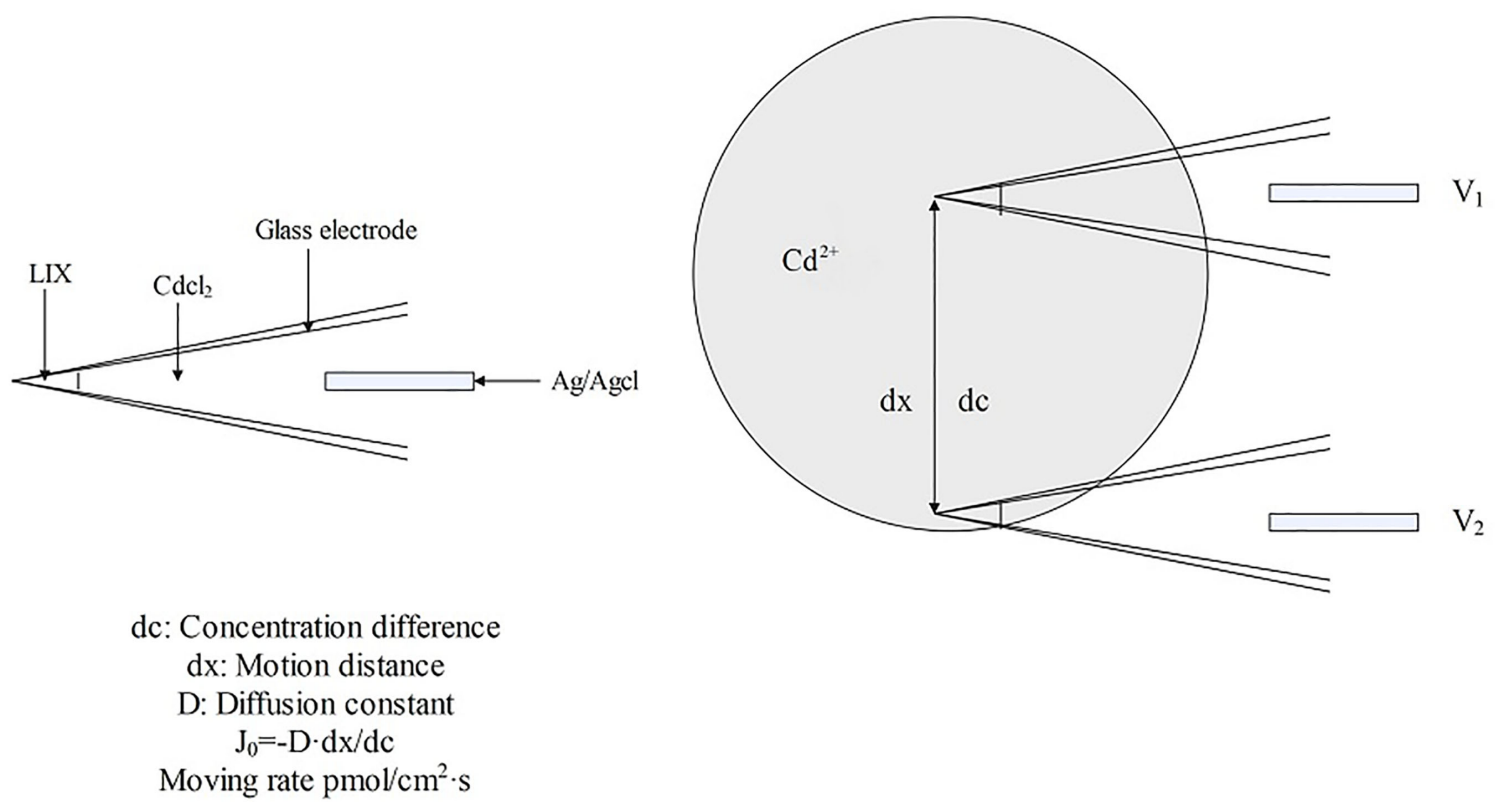
Schematic diagram of Cd^2+^ detection by NMT.

Non-invasive micro-test technology (NMT) is a microelectrode technology for the real-time, label-free detection of the flow rate and the influx or efflux of molecules and ions into the organisms (Yuan et al., 2021). NMT consists of a selective microelectrode system (SMS), signal amplification, microscopic imaging, 3D motion, and data processing systems. The SMS comprises a glass microsensor, Ag/AgCl electrode, electrolyte, and liquid ion exchanger (LIX). The selectivity is achieved through the specific recognition of ions by organic compounds in the large neutral molecular carriers within the LIX, this is essential for NMT. The selective microelectrode is moved back and forth between high and low electrochemical potentials carried by charged particles, measuring the potential value at each point obtaining the potential difference (AV) between the two points. The potential concentration calibration curve of the electrode was employed to measure the differences in the ion concentrations between these two points. The rate of ion movement was computed using Fick’s First Law of Diffusion and Nernst’s Equation (**Figure 10**). NMT can perform 3D measurements of the samples *in situ* with high sensitivity and spatial resolution without causing damage or requiring marker sampling. It is simple to operate and can detect the dynamic indicators of bioactivities of plants (Yuan et al., 2021; Wang et al., 2023). It can detect 50 types of metallic molecules and ions, including H^+^, Ca^2+^, Na^+^, K^+^, Cl^-^, Cd^2+^, Cu^2+^, and NH_4_^+^ (Mugnai et al., 2012; Mardones et al., 2018; Wu et al., 2019; Lan et al., 2020; Zhang et al., 2020; Di et al., 2021; Hou et al., 2021). As the rate of NMT-based determination is robust, it enables the investigation of the flow characteristics of metal ions (mainly in roots); it can be used to screen species as low or hyperaccumulators and explore the enrichment characteristics of plants; and it provides technical support for hyperaccumulator-based remediation of contaminated soils, plant nutrition regulation, and plant physiology research (Han et al., 2022).

NMT is a handy tool for studying ion flow, signal transduction, and functional genes in plants under HM-induced stress. It can monitor the dynamic flow of HM ions in and out of plants in real-time, which is conducive to investigating the mechanism of tolerance to HMs, the toxic effects of HMs, and the mechanism by which hyperaccumulators absorb and transport HM ions (Liu et al., 2020). The scanning ion selective electrode technology (SIET) and microelectrode ion flux estimation technology (MIFE) are commonly used for measuring ion currents in plants (Mardones et al., 2018; Hou et al., 2021; Sun et al., 2021; Sun et al., 2021; Deng et al., 2022). The inflow of Cd^2+^ at 300 µm around the root tips of *Bidens pilosa* L. plants was reduced when treated with 16 mM Ca^2+^, 8 mM Mg^2+^, 8 mM SO_4_^2-^, or 18 mM K^+^ as detected by NMT, indicating that treatment at a large concentration of nutrient ions inhibited the absorption of Cd^2+^ (Wang et al., 2023). Using the NMT method, a peak in the net Cd^2+^ flux was observed at a distance of 100 µm from the surface of the root tips of *Amaranthus hypochondriacus* L., a plant famous for its ability to accumulate large quantities of this metal ion (Han et al., 2023). Furthermore, fluorescent labeling revealed that Ca^2+^ channel blockers conspicuously suppressed Cd^2+^ influx compared to other blockers, suggesting that Ca^2+^ channels serve as primary pathways for Cd entry through the roots (Han et al., 2023). In a similar observation, Cd primarily entered *Sedum plumbizincicola* through Ca^2+^ channels; the net flux of Cd^2+^ altered from inflow to outflow as the concentration of Ca^2+^ increased (Li et al., 2017b). NMT detected a significant reduction in the flow of Cd^2+^ in the root hair area of rice plants when NH_4_^+^ was added, indicating that it reduced the influx of Cd and, therefore, is beneficial for cultivating rice with lower levels of pollutants (Wu et al., 2018). The influx and efflux of ions from plant cells were detected by NMT, providing the most intuitive and accurate data for studying plant stress resistance mechanisms and understanding the relationship between ion/molecular flow and specific cellular functions (Han et al., 2022).

### 3.5 Omics approaches for HM response and tolerance in plants

Plant reactions to HM toxicity are dependent on regulation by molecular factors (Raza et al., 2022). Deciphering HM toxicity and subsequent molecular, cellular and physiological responses is regarded to be a challenging, arduous and complex work (Mondal et al., 2022). A diverse range of biotechnologies are being applied to better understand and explore the pathways and mechanisms involved in plant responses and tolerance for HM toxicity (Raza et al., 2022). With the advancement of science and technology, the emergence of omics has provided a valuable terrace to investigate the physiological, biochemical, metabolical and molecular activities of HM-treated plants and cells. Well-established genomics technologies like genomics, transcriptomics, proteomics, metabolomics, ionomics, and other high-throughput technologies are valuable in elucidating plant stress responses and tolerance to HM. Through a comprehensive analysis of a large amount of omics data, the fundamental state of genes, mRNAs, proteins, metabolites, and elemental compositions in plants could be ascertained to fully explain the molecular mechanisms underlying the interaction between plants and HMs. The data gained will contribute to the enhanced research on plant stress tolerance and can be used in breeding and engineering schemes to breed plants with new and desirable agronomic traits and hyperaccumulators (Singh et al., 2016).

Analysis of genomics-based data aids in identifying genes, regulators, enzymes, signaling and other molecular factors involved in the HM uptake, transportation, resistance, and tolerance mechanisms of plants (Anjitha et al., 2023). DNA mismatch repair (MMR), targeted induced local lesions in genomes (TILLING), genome-wide association studies (GWAS), and clustered regularly interspaced short palindromic repeats (CRISPR/Cas9) are some of the genomic methods that recognize genes are correlated with the interactions between plant and metal (Al-Khayri et al., 2023). GWAS study in *Medicago sativa* subjected to Cd stress reveals that response features are polygenic, involving several quantitative loci, and that stress-related UDP-glycosyltransferases, genes ATP-binding cassette transporters, *P*-type ATP transporters genes, and oxidative stress response genes are associated with Cd tolerance (Paape et al., 2022). In barley, under Cd stress, the genetic structure and genetic regulation study by QTL analysis revealed that antioxidant enzymes, stress signaling proteins and metal ion transporters showed different categories of InDels and SNPs in the QTL regions between the parents, which probably be critical for responding to Cd stress (Derakhshani et al., 2020). The ABC transporters detected are potential candidate genes that can be explored for raising of Cd-tolerant barley varieties (Derakhshani et al., 2020).

Transcriptomic profile can reflect the transcriptome differences among different cultivars, developmental stages, and tissues of plants under different environmental conditions, which provides us with further understanding and analysis of the responses under specific physiological states or stress conditions (Jamla et al., 2021). Transcriptomic analysis of *Calotropis gigantea* on exposure to Cd demonstrated that the leaves mitigated Cd stress by activating a variety of Cd detoxification processes such as cell wall remodeling, antioxidant systems, metal chelation, and the upregulation of genes related to the metal transporters (Yang et al., 2022). Using a gene co-expression network, the researchers was identified that *BnaCn.ABCC3* and *BnaC3.ABCC4* in Brassica are a pivotal factors participating in the storage of PC-Cd complexes in vacuolar (Zhang et al., 2019). The amount of genes participating in survival mechanisms and metabolic pathways would have been regulated by HM stress. Regulatory genes (e.g., TF) together with these functional genes are critical for the regulation of many stress-responsive genes, and will ultimately form gene networks (Jamla et al., 2021). In *Arabidopsis*, MYB4 TF modulates Cd tolerance by ameliorating the anti-oxidant defense system and enhancing the expression of the promoter regions of phytochelatin synthase 1 (*PCS1*) and metallothionein 1C (*MT1C*) (Agarwal et al., 2020).

The new insights provided by proteomics into stress-induced proteins and their involvement in mitigating HM toxicity is a powerful tool (Raza et al., 2022). It has also been used to compare changes in proteins at the organelle, cell, tissue and organ levels, and to provide an understanding of protein behavior under diverse stress situations, including HM stress (Singh et al., 2016). By 2-DE analysis, 18 proteins involved in Cd tolerance were discovered in the leaves of two tobacco genotypes, Gui Smoke 1 (Cd-sensitive) and Yunnan Smoke 2 (Cd-tolerant) (Ghatak et al., 2017). Proteomic analysis (LC-MS/MS) of Se-stressed pepper seedlings showed that 172 proteins were up-regulated and 28 proteins were down-regulated, and several heat shock proteins (HSPs), which help to cope with Se stress, were identified in the Se stress-induced differentially expressed proteins (DEPs) of pepper (Zhang et al., 2019).

Under stress conditions, plants become more focused on coping with adversity stress and survival, and regulate their growth and developmental processes. This leads to changes in the pattern of gene expression (or transcriptional reprogramming), which then causes changes in the concentration, type, and number of metabolites produced (Jamla et al., 2021). Metabolomics helps to understand the complex metabolic activities and differences in regulated metabolites produced by plants under different conditions including HM stress (Raza, 2022). In Cd-stressed tobacco plants, metabolomics investigation indicated that 76 metabolites in the leaves and 150 metabolites were in the roots individually. It is found that metabolites were in considerable abundance in the biosynthesis of amino acids, arginine and proline, flavone and flavonol, and nicotinate and nicotinamide (Zou et al., 2022). In *Calendula officinalis* plant exposed to Cd stress, the phenomena of the contest between specialized metabolites (triterpenoids) and general metabolites (sterols) were noted in the culture of plant roots and hairy root (Rogowska et al., 2022).

Plants need a suitable amount of minerals to maintain normal vital activities. The application of ionomics enables an in-depth understanding of the nutrient and trace element makeup of plants, explores the activities and mechanisms of plant absorption, storage, and assimilation, and provides new ideas for illuminating the response of plants to HMs (Raza et al., 2022). It has been shown that plants subjected to HM-induced stress can be adapted to unfavorable conditions via modulating elemental composition and content in the ionome (Mleczek et al., 2018). Researchers used transcriptomic and ionomic mapping to analyze the mechanisms of mitigating As(III) force in rice and found that ionome was significantly correlated with transcriptome in the aerial part of rice (Xiao et al., 2021). Upregulation of transporter genes associated with transmembrane transport and ion binding associated with nutrient elements was found, showing that plants tend to be more inclined to transport more nutrients, like ammonium, potassium and phosphate, in response to As(III) toxicity (Xiao et al., 2021).

The accurate selection of HM detection and analysis methods is crucial for explaining the stress caused by HMs in plants. By conducting a comprehensive analysis of the different, commonly used methods for HMs in plants, the application criteria and characteristics of each method were obtained, systematically compared, and summarized (**Table 2**).

**Table 2:** Comparison of HM detection and analysis methods commonly used in plants.

Method	Element detection range	linear range	sensitivity	Speed of analysis	Sample Preparation	flammable gas	isotopic analysis	Simultaneous multi-element or multi-ion analysis	operation	cost	LOD	major interference	reference
ICP-MS	wide	widest	Highest	Fast	simple	NO	YES	YES	simple	High	ppt	spectral interference	(Fu et al., 2018; Wilschefski and Baxter, 2019; Theiner et al., 2020; Penanes et al., 2022)
AAS	wide	narrow	High	slow	complex	YES	NO	NO	simple	Low	ppb	Matrix Interference	(Suquila et al., 2019; Eskina et al., 2020; Li et al., 2021; Zvěřina et al., 2023)
AFS	narrow	wide	Higher	slow	complex	NO	NO	YES	simple	Low	ppb	spectral interference	(Yogarajah and Tsai, 2015; Lancaster et al., 2019; Li et al., 2019; Xia et al., 2019; Liu et al., 2020; Peng et al., 2022)
XAS	wide	***	Highest	Fast	simple	NO	NO	YES	simple	High	***	spectral interference	(Newville, 2014; Mastelaro and Zanotto, 2018; Tang et al., 2023)
XRF	wide	wide	High	Fast	simple	NO	NO	YES	simple	Low	ppb	spectral interference	(Chuparina and Aisueva, 2011; McGladdery et al., 2018; Bamrah et al., 2019)
LA-ICP-MS	wide	widest	Highest	Fast	simple	NO	YES	YES	complex	High	ppt	spectral interference	(Davison et al., 2023; Janovszky et al., 2023)
NMT	wide	***	Highest	Fast	complex	NO	NO	YES	simple	High	***	sample status	Yuan et al., 2021; Wang et al., 2023)

*** means that no relevant information is given in the literature.

## 4 Conclusions and prospects

With rapid urbanization and industrialization, HM pollution has become a major environmental problem. HMs released in the environment are absorbed and accumulated from plants, which are transmitted to humans along the food chain, causing grave adverse impacts on the life and health of plants and humans. Therefore, it is crucial to propose solutions that effectively control the content of HMs by studying the mechanisms of translocation, uptake, distribution, and storage of HMs in plants and identifying their chemical forms. This paper reviews the technical methods applied to study HMs in plants during recent years and summarizes the principles, advantages, disadvantages, and applications for these approaches.

ICP-MS and AAS are widely used to detect HM content in plants, both of which have the advantages of high sensitivity, a low detection limit, and high accuracy. Compared to AAS, ICP-MS has higher operating costs; however, it offers convenient and fast detection, lesser interference, simple sample preparation, and the ability to analyze multiple elements and their isotopes (Pei et al., 2023). These advantages meet the needs of large-scale detection, necessitating researchers with an expectation of obtaining additional information to choose ICP-MS more frequently. Therefore, designing and developing portable instruments based on ICP-MS and AAS-based multi-element analyses have become a priority. AFS and XAS are primarily used to study the chemical form of HMs in plants. AFS has a good selectivity, but its limited range of detecting elements severely restricts its application. XAS is preferred more because of its low maintenance cost and ability to provide more information, such as the type of coordinating atoms, coordination number, atomic spacing, which aligns with daily laboratory use. In recent years, XRF and LA-ICP-MS have become the primary methods often applied to the spatial distribution of HMs in plants. LA-ICP-MS, with its simple operability and instrumentation, has been widely used for studying the tissue distribution of HMs in plants. This technology can provide a basis for studying the mechanisms of HM transport and the function and localization of transport proteins by analyzing the distribution of HMs. Although SR-XRF requires large-scale instrumentation, the combined use of SR-XRF and XAS is often chosen for analyzing the chemical for and tissue distribution of HMs in plants (Bai et al., 2019; Li et al., 2020). NMT has demonstrated outstanding performance in monitoring the flow speed and direction of small molecules and ions in organisms and has rapidly developed in recent years. The difficulty in fabricating and preserving selective microelectrodes has limited their application to HM-related studies (Han et al., 2022). The rise and application of molecular biology and omics technologies have opened a new chapter in studying HMs in plants. Omics can determine the interactions between plants and HMs by obtaining metabolomics, transcriptomics, genomics, ionomics, and proteomics information on plants subjected to HM-induced stress. Molecular biology techniques, such as gene cloning and editing, investigate the function of proteins and genes and the response mechanisms of plants to HMs and provide theoretical guidance for improving the tolerance and resistance of plants to HMs.

In recent years, ICP-MS, XAS, LA-ICP-MS, NMT, omics, and molecular biology techniques have been increasingly applied in HM-related research in plants, providing technical support for studying the uptake, transport and distribution, accumulation and storage, and detoxification of HMs in plants. Using omics and molecular biology methods, the mechanisms of plant response to HM-induced stress were analyzed, and the plant varieties with HM resistance or low HM accumulation were screened. Furthermore, regarding environmental concerns, future research may focus on screening and breeding hyperaccumulating plant cultivars to remediate contaminated soils. The use of traditional methods for detecting HMs is maturing. Regarding HM detection technology, low-cost operability, and portability have become the focus of attention and application in laboratory research. Therefore, simplifying the operability of instruments, expanding their detection range, and reducing the costs of different detection technologies remain the main directions of progress. The detection technology of HMs in plants should be continuously refined and improved to develop it towards integration, efficiency, and high adaptability.

## Author contributions

SH: Writing – original draft. YN: Writing – original draft. LX: Writing – original draft. ZL: Writing – review & editing. XS: Writing – review & editing. MD: Writing – review & editing. WH: Writing – review & editing.
