# Supplementary material for: Research progress of the detection and analysis methods of heavy metals in plants
Source: Front Plant Sci. 2024 Jan 31;15:1310328. doi: 10.3389/fpls.2024.1310328 (PMC10867983; doi:10.3389/fpls.2024.1310328)
Supplement: Supplementary file 1 [file Table_1.docx]

Supplementary Material

**Non-standard abbreviations**

Non-Standard Abbreviations list

| **Abbreviations** | **Full name** | |
| --- | --- | --- |
| HM | Heavy metal |  |
| ICP-MS | Inductively coupled plasma mass spectrometry |  |
| AAS | Atomic absorption spectrometry |  |
| AFS | Atomic fluorescence spectrometry |  |
| XAS | X-ray Absorption Spectroscopy |  |
| XRF | X-ray fluorescence spectrometry |  |
| LA-ICP-MS | Laser ablation-inductively coupled plasma-mass spectrometry |  |
| NMT | Non-invasive Micro-test Technology |  |
| ROS | Reactive oxygen species |  |
| Al | Aluminum |  |
| PCs | Phytochelatins |  |
| ZIPs | Zn-regulated transporter, iron-regulated transporter-like Proteins |  |
| HMA | HM-associated domain |  |
| IRT | Iron-regulated transporter |  |
| NRAMP | Natural resistance-associated macrophage protein |  |
| YSL | Yellow stripe 1-like family |  |
| AsIII | Arsenite |  |
| AsV | Arsenate |  |
| FRO | Ferric reductase/oxidase |  |
| XANES | X-ray absorption near-edge spectroscopy |  |
| HPLC-ICP-MS | High-performance liquid chromatography/inductively coupled plasma mass spectrometry |  |
| DMA | Dimethyl arsenic acid |  |
| MMA | Methyl arsenic acid |  |
| PhHg | Phosphorus mercury |  |
| EtHg | Ethyl mercury |  |
| MeHg | Methyl mercury |  |
| SEM | Scanning electron microscopy |  |
| SR | Synchrotron radiation |  |
| His | Histidine |  |
| Cys | Cysteine |  |
| GSH | Glutathione |  |
| MTs | Metallothioneins |  |
| γ-Glu-Cys | γ-glutamyl-cysteine |  |
| H_2_O_2_ | Hydrogen peroxide |  |
| MDA | Malondialdehyde |  |
| G6PDH | Glucose-6-phosphate dehydrogenase |  |
| RF | Radio frequency |  |
| WHO | World Health Organization |  |
| ICP-MS-MS | Inductively coupled plasma-Tandem mass spectrometry |  |
| SP-ICP-MS | Single-particle inductively coupled plasma mass spectrometry |  |
| LC-ICP-MS | Liquid chromatography-inductively coupled plasma mass spectrometry |  |
| ESI-MS/MS | Electrospray ionization-tandem mass spectrometry |  |
| CVAAS | Cold vapor generation atomic absorption spectrometry |  |
| HGAAS | Hydride generation atomic absorption spectrum |  |
| GFAAS | Graphite furnace atomic absorption spectrometry |  |
| FAAS | Flame atomic absorption spectrophotometry |  |
| CVAFS | Cold vapor atomic fluorescence spectrometry |  |
| HGAFS | Hydride generation atomic absorption spectrum |  |
| UDUE | Ultrasonic-assisted enzyme digestion |  |
| LC-HGAFS | Liquid chromatograph-hydride generation atomic fluorescence spectrometry |  |
| HPLC-HG-AFS | High-performance liquid chromatography and hydride generation atomic fluorescence spectrometry |  |
| LC-AFS | Liquid chromatograph-atomic fluorescence spectrophotometry |  |
| HG-UV-AFS | Hydride generation novel ultraviolet atomization atomic fluorescence spectrometry |  |
| MeHgH | Methyl Hg^+^ hydride |  |
| MeHg | Methyl Hg^+^ |  |
| RP-HPLC-HG-AFS | Reversed-phase high performance liquid chromatography and hydride generation atomic fluorescence spectrometry |  |
| EXAFS | Extended X-ray absorption fine structure spectroscopy |  |
| EDXRF/EDX | Energy scattering X-ray fluorescence spectroscopy |  |
| WDXRF/WDX | Wavelength scattering X-ray fluorescence spectroscopy |  |
| XPS | X-ray photoelectron spectroscopy |  |
| SRXRF | Synchrotron radiation X-ray fluorescence spectroscopy |  |
| ppb | part per billion |  |
| μ-XRF | Microscopic X-ray fluorescence |  |
| SR-μXRF | Synchrotron radiation microscopic X-ray fluorescence |  |
|  |  |  |
| LXD | Laser exfoliation device |  |
| SMS | Selective microelectrode system |  |
| LIX | Liquid ion exchanger |  |
| AV | Potentiometric difference |  |
| SIET | Scanning ion-selective electrode technology |  |
| MIFE | Microelectrode ion flux estimation |  |
| MMR | Mismatch repair |  |
| TILLING | Targeted induced local lesions in genomes |  |
| GWAS | Genome-wide association studies |  |
| CRISPR/Cas9 | Clustered regularly interspaced short palindromic repeats |  |
| PCS1 | Phytochelatin synthase 1 |  |
| MT1C | Metallothionein 1C |  |
| HSPs | Heat shock proteins |  |
| DEPs | Differentially expressed proteins |  |
